# Molecular Signaling Pathways in Depression and Neuroinflammation: Focus on New Pathophysiological Proteins and Therapeutic Compounds

**DOI:** 10.3390/antiox15091195

**Published:** 2026-09-18

**Authors:** Hyeong Jae Kim, Jeong-Hee Kim, Young-Don Son, Jong-Hoon Kim, Jeong Hee Hong

**Affiliations:** 1 Department of Physiology, Lee Gil Ya Cancer and Diabetes Institute, Gachon University College of Medicine, 155 Getbeolro, Yeonsu-gu, Incheon 21999, Republic of Korea; fgfgfg1125@gachon.ac.kr; 2 Institute of Human Convergence Health Science, Gachon University, 191 Hambakmoe-ro, Yeonsu-gu, Incheon 21936, Republic of Korea; jhkim1104@gachon.ac.kr; 3 Neuroscience Research Institute, Gachon University, 21 Namdong-daero 774 beon-gil, Namdong-gu, Incheon 21565, Republic of Korea; ydson@gachon.ac.kr; 4 Department of Biomedical Engineering, College of IT Convergence, Gachon University, 1342 Seongnam-daero, Sujeong-gu, Seongnam-si 13120, Republic of Korea; 5 Department of Psychiatry, Gachon University College of Medicine, Gachon University Gil Medical Center, 21 Namdong-daero 774 beon-gil, Namdong-gu, Incheon 21565, Republic of Korea

**Keywords:** depression, neuroinflammation, oxidative/nitrosative stress, calcium

## Abstract

Neuroinflammation is a multifaceted process in which specific biomolecules (such as pro-inflammatory cytokines and signaling proteins) act as primary mediators of neuropsychiatric disorders, including depression. Despite growing evidence, the precise interconnected cascades within these neuroinflammatory signaling pathways and their regulatory proteins remain to be fully elucidated, which presents a significant unmet need for targeted therapeutic strategies with clear mechanistic validation. Accordingly, the present review analyzes the impact of neuroinflammation on the pathophysiology of depression in various rodent models. We explore the regulatory mechanisms by which factors such as oxidative stress, calcium signaling, and inflammasome-mediated inflammation affect neurotransmitter synthesis and neuronal integrity. Furthermore, we elucidate the therapeutic potential of synthetic compounds and naturally derived agents targeting these specific molecular pathways. Based on a systematic literature search, this review focuses exclusively on pharmacological agents with clearly defined molecular mechanisms and rigorous depression-associated in vivo validation, thereby providing promising therapeutic strategies for the restoration of neurobiological homeostasis.

## 1. Introduction

Major depressive disorder (MDD) affects nearly 3.9% of the global population and represents the most critical paradigms due to their unparalleled disease burden with structural impact on the brain, and profound clinical severity [1,2]. While alterations in monoaminergic neurotransmission have long been implicated in the pathophysiology of MDD, accumulating evidence suggests that neuroimmune dysregulation is an important contributor to depressive pathophysiology, prompting a shift toward broader neurobiological mechanisms [3]. Central to this evolving perspective is neuroinflammation, a multifaceted process that responds to injuries, infections, and pathological events. Characterized by the involvement of diverse biomolecules and cellular responses, it is posited not as a primary etiology, but rather as a potential contributing or modulating factor in the pathophysiology of neuropsychiatric disorders, including depression, schizophrenia, and bipolar disorder [3,4,5,6].

To understand neuropsychiatric disorders such as depression, various pathophysiological mechanisms have been proposed and studied. In particular, various studies have emphasized that the onset of neuroinflammation is promoted by the production of reactive oxygen species (ROS) and mobilization/circulation of pro-inflammatory mediators, notably interleukin (IL)-1β, IL-6, and tumor necrosis factor (TNF)-α [7,8,9]. These inflammatory molecules act as pivotal orchestrators, initiating downstream signaling pathways such as mitogen-activated protein kinase (MAPK) pathways, particularly the extracellular signal-regulated kinase (ERK) cascade within glial cells, thereby promoting pro-inflammatory cytokine production and sustaining neuroinflammatory responses [9,10,11].

The sustained activation of inflammatory signaling pathways leads to profound transcriptional and functional remodeling of microglia, characterized by enhanced inflammatory signaling and altered interactions with neurons and astrocytes [12,13,14,15]. This microglial remodeling promotes excessive oxidative/nitrosative stress and the maturation of the inflammasome complex, further augmenting the feedback loop of inflammation [16]. Moreover, several studies have reported that oxidative/nitrosative stress leads to lipid peroxidation, reduced glutathione (GSH) levels, DNA damage and an attenuated enzymatic reduction of oxidative stress, all of which are involved in the pathophysiology of depression and anxiety [17,18,19,20]. Crucially, these inflammatory molecules are active participants in disrupting the structural integrity of the blood–brain barrier (BBB), thereby inducing enhanced permeability to peripheral immunocytes and disrupting the homeostatic environment necessary for neuronal health [16].

**Figure 1:**
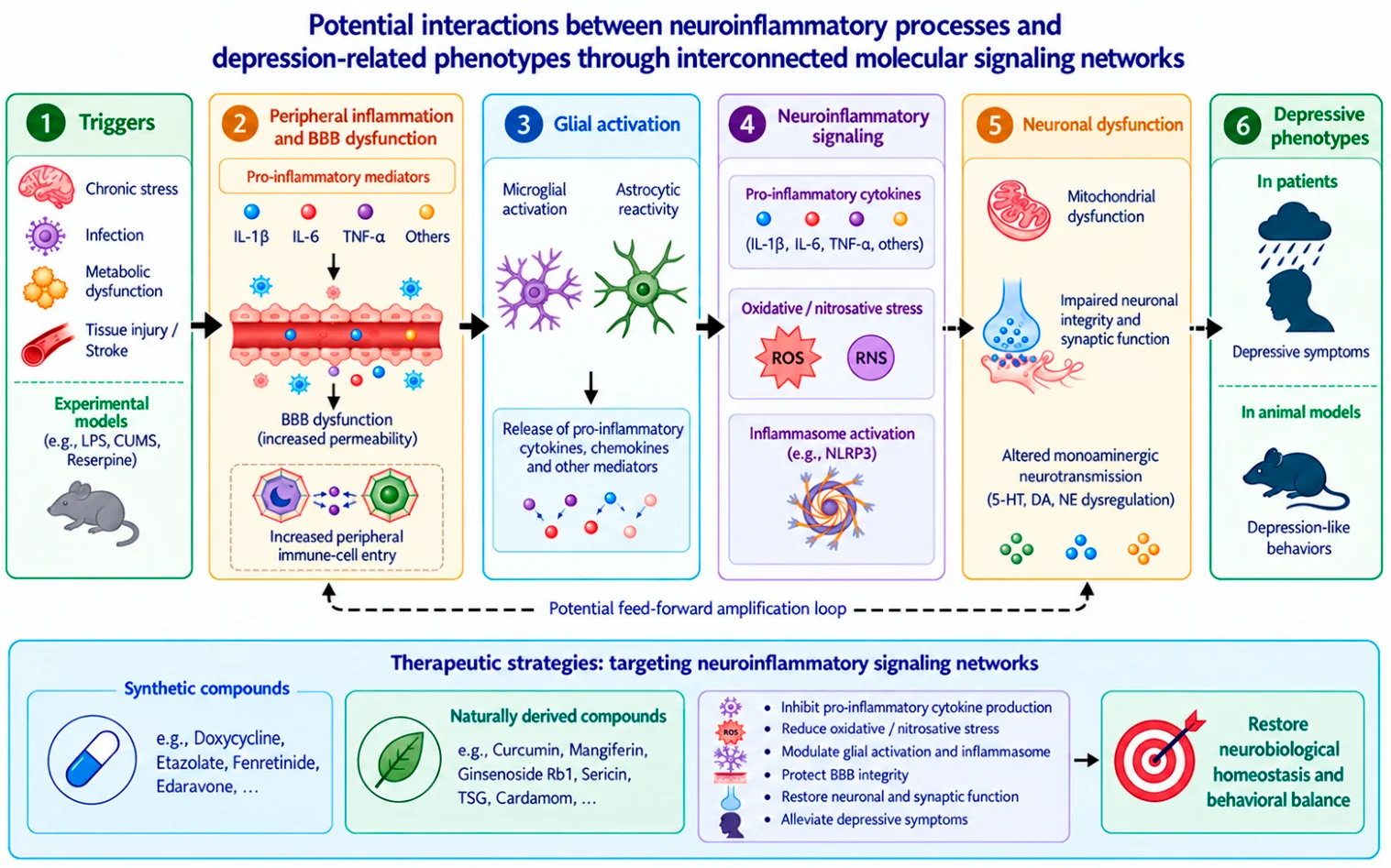
Schematic illustration of interconnected neuroinflammatory signaling pathways contributing to the pathophysiology of depression and their potential therapeutic targets. Neuroinflammation is initiated by diverse clinical and pathological factors, including chronic stress, infection, metabolic dysfunction, and tissue injury, as well as by experimental inducers commonly used in animal models. These stimuli promote peripheral inflammatory responses and BBB dysfunction, facilitating glial activation and the release of pro-inflammatory mediators. Sustained neuroinflammatory signaling, characterized by excessive cytokine production, oxidative/nitrosative stress, and inflammasome activation, contributes to neuronal and synaptic dysfunction, ultimately promoting depressive symptoms in patients and DLBs in animal models. Therapeutic interventions targeting these interconnected neuroinflammatory pathways—including synthetic compounds and naturally derived agents—may attenuate inflammatory responses, preserve neuronal homeostasis, and restore behavioral function. Solid arrows indicate established relationships, whereas dotted arrows indicate potential or indirect relationships rather than definitive causal links. Abbreviation: DLBs, depression-like behaviors; BBB, blood–brain barrier; IL, interleukin; TNF-α, tumor necrosis factor-α; ROS, reactive oxygen species; RNS, reactive nitrogen species; NLRP3, NOD-like receptor family pyrin domain containing 3; 5-HT, serotonin; DA, dopamine; NE, norepinephrine; LPS, lipopolysaccharide; CUMS, chronic unpredicted mild stress; TSG, total saponins of *Panax ginseng*.

In the absence of homeostatic control, the feed-forward loop of oxidative damage and inflammasome-mediated neuroinflammation not only impairs neuronal integrity but also is increasingly recognized to contribute to the manifestation of depressive symptoms in patients and depression-like behaviors (DLBs) in animal models (Figure 1). The present review, therefore, provides a focused evaluation of the key biomolecules and cytokines that constitute this neuroinflammatory milieu, highlighting their potential roles in modulating depression-related pathophysiology. By evaluating established and emerging evidence regarding their mechanisms of action, we highlight how these discrete molecular signatures converge to modulate the pathophysiology of depression in various stress- or agent-induced experimental models of the depressive disorder. Furthermore, we elucidate the therapeutic potential of diverse synthetic compounds and naturally derived agents that target these specific molecular nodes, offering promising targets and expanded strategies to attenuate the neuroinflammatory drive and restore neurobiological homeostasis and behavioral equilibrium.

## 2. Methodology and Inclusion Criteria

This review comprised studies retrieved from PubMed and focused on the effects of various factors, such as oxidative stress and calcium (Ca^2+^) signaling, on neuroinflammation and depression. In particular, we focused on depression models, signaling proteins, and the potential of pharmacological agents (synthetic and natural-derived agents) as treatment strategies. Furthermore, the current review explored the relationship between diverse disease models and depression, but also elucidated a molecular regulatory mechanism underlying neuroinflammation-driven depressive disorders.

### 2.1. Search Strategy and Literature Selection

A literature search was conducted on PubMed for articles published between 1972 and 2026. The complete search string was structured as follows: oxidative stress or nitrosative stress, Ca^2+^, neuroinflammation, depression, neuronal apoptosis, and hyperglycemia.

### 2.2. Inclusion/Exclusion Criteria and Screening Process

Studies were selected according to predefined eligibility criteria.

#### 2.2.1. Inclusion Criteria

(1) Study design and models: Peer-reviewed original research articles utilizing in vivo animal models of depression and disease-related models presenting depression-like behavioral or neuroinflammatory outcomes;
(2) Signaling pathways: Studies evaluating key regulatory proteins that act as initiators and amplifiers of inflammation, mediators of oxidative stress and cell death, or regulators of ion dyshomeostasis, representing core convergence points of anti-depressant efficacy;
(3) Pharmacological interventions: Studies investigating pharmacological agents (synthetic and natural-derived) sharing a common therapeutic axis—specifically, the attenuation of neuroinflammatory cascades, suppression of cellular stress pathways (e.g., ER stress and ferroptosis), and the restoration of microglial homeostasis.

#### 2.2.2. Exclusion Criteria

(1) In vitro-only studies without accompanying in vivo validation;
(2) Non-peer-reviewed publications or conference abstracts.

#### 2.2.3. Screening Process

Title, abstract, and full-text screening was performed independently by four reviewers. Discrepancies were resolved through group discussion and consensus. After applying the eligibility criteria, the selected original studies were included in this review.

## 3. Emerging Candidate Signaling Proteins in Depression and Neuroinflammation

This section explores the intracellular components induced by neuroinflammation to elucidate the molecular mechanisms underlying depression. In this context, we highlight a diverse array of emerging candidate signaling molecules categorized by their primary pathological axes: initiators and amplifiers of the inflammation [NOD-like receptor (NLR) pyrin domain-containing proteins (NLRPs) and sterile α and TIR motif containing 1 (SARM1)], oxidative stress and induction of cell death [peroxiredoxins (Prdxs), nitric oxide synthase (NOS), and glutathione peroxidase 4 (GPx4)], and ion dyshomeostasis [Na^+^, K^+^-ATPase (NKA), sarco/endoplasmic reticulum Ca^2+^-ATPase 2 (SERCA2), synaptojanin-2-binding protein (SYNJ2BP), and endophilin A1 (EPA1)]-encompassing additional regulatory factors that collectively drive synaptic dysfunction and neuronal impairment within the depressive brain. To facilitate understanding, these targets and their preliminary supporting evidence in depression and disease-related models are summarized in Table 1.

### 3.1. The Initiators and Amplifiers of the Inflammation

#### 3.1.1. NOD-like Receptor Pyrin Domain-Containing Protein

NLRs are intracellular pattern recognition receptors that monitor various pathogen-associated molecular patterns and damage-associated molecular patterns and are able to form multimeric inflammasome complexes [21,22]. In the central nervous system, NLRP1 is principally expressed within cortical and spinal neurons, as well as oligodendrocytes, with lower levels detected in other glial populations [23]. The NLRP1 inflammasome promotes activation of caspase (Casp)1, and this interaction leads to pro-Casp1 auto-cleavage, forming biologically functional p10/p20 heterodimers [24]. Subsequently, activation of Casp1 results to maturation and secretion of pro-IL-18 and pro-IL-1β. Consequently, activation of IL-18 and IL-1β mediates innate and adaptive immune systems [25]. Among NLRP isoforms, NLRP3, known as cryopyrin and NALP3, is the best characterized component of inflammasome [21] which is formed by stimulation of toll-like receptor (TLR) activation, cytokine, and others [26]. NLRP3 expression is founded in neutrophils, monocytes, and dendritic cells, which are thought to be high producers of IL-1β [27]. Specifically, activation of NLRP3 inflammasome is induced through enhanced the generation of ROS and thioredoxin-interacting protein (TXNIP) by chronic hyperglycemia; subsequently, the ROS/TXNIP axis promotes the activation and secretion of Casp1-dependent IL-1β [28,29]. Moreover, hyperglycemia enhances permeability of the blood–brain barrier and neutrophil infiltration and subsequently induces brain ischemic injury [30]. Streptozotocin (STZ), known as a chemical for inducer of type 1 diabetes mellitus, affects insulin-producing pancreatic β-cells through its toxic effect and has been demonstrated to mimic diabetes-associated depression mouse models [31,32,33,34,35]. To mimic the hyperglycemic mouse model, microglia is stimulated by high glucose [36]. DLBs, NLRP3 activation, and neuroinflammation are ameliorated by the pharmacological inhibition of NLRP3 using MCC950 in the hippocampal region of a STZ-elicited hyperglycemic rodent model [36]. Neuroinflammation is enhanced through activated nuclear factor kappa-light-chain-enhancer of activated B cells (NF-κB)-NLRP3 pathway and phosphorylated double-stranded RNA-dependent protein kinase (PKR)-TXNIP-ROS-NLRP3 signaling by treatment with high glucose in mouse microglia [36].

#### 3.1.2. Sterile α and TIR Motif Containing 1

Sterile α and TIR motif containing 1 (SARM1), as a NAD+ hydrolase, possesses crucial functions in immune responses and neuronal degeneration [37]. A study recently showed that knockdown of SARM1 induces the phosphorylated form of c-Jun N-terminal kinase (JNK) and subsequently induces DLBs and synaptic impairment in mice [38]. Genetic and pharmacological deletion of SARM1 exacerbates neuroinflammation through the activation of the JNK/stimulator of interferon genes (STING)/TANK-binding kinase (TBK)1 and NLRP3 signaling pathways in both hippocampal tissues and neuronal cells [38]. Notably, these pro-inflammatory effects and the concomitant production of ROS are attenuated by the JNK inhibitor SP600125, indicating that the SARM1-deficiency-elicited inflammatory response is mediated via the JNK/STING/TBK1/NLRP3 signaling axis [38].

### 3.2. Oxidative Stress and Induction of Cell Death

#### 3.2.1. Peroxiredoxins

Peroxiredoxins (Prdxs) are a family of conserved antioxidant enzymes that scavenge excessive ROS and oxidative stress [39,40]. A recent study addressed that LPS-elicited depression models exhibit significant behavioral and molecular alterations [41]. Systemic administration of LPS not only triggers depression-like symptoms but also exacerbates neuroinflammation and ROS production within the hippocampal region and microglial cells. In response to the amplified oxidative stress, the expression levels of Prdxs members, including Prdx 1, 2, 4, and 5, are significantly upregulated, suggesting a localized antioxidant defense mechanism in the LPS-stimulated neuroenvironment [41].

#### 3.2.2. Nitric Oxide Synthase

Several studies report that pro-inflammatory cytokines, which interact with various pathophysiologic targets concerning depressive disorder, including dysfunction of neurotransmitter metabolism, neuroendocrine function, and synaptic plasticity [42,43], are upregulated in MDD patients [42,44]. NOSs have three isoforms, comprising neuronal (nNOS), inducible (iNOS), and endothelial NOS (eNOS), and possess biological functions, including regulation of vascular homeostasis, neurotransmission, and immunological surveillance [45]. Among them, iNOS mediates NO overproduction, thereby leading to neurotoxicity [46,47]. Moreover, NO is produced through L-arginine oxidation by NOS in mammalian cells, and functions as a pivotal regulatory factor of diverse physiological homeostasis in the immune, cardiovascular, and nerve systems [46]. Neuroinflammation is attenuated through NO-cGMP signaling via inhibition of NOS and phosphodiesterase (PDE) 5 by treatment with N^ω^-nitro-L-arginine methyl ester hydrochloride (L-NAME), aminoguanidine or sildenafil, respectively, thereby decreasing DLBs in the hippocampal region of LPS-elicited rodent models of depression [48]. Moreover, antioxidant effects are enhanced through increased GSH levels by treatment with L-NAME, aminoguanidine or sildenafil in the hippocampal region of LPS-elicited rodent models of depression [48].

#### 3.2.3. Glutathione Peroxidase 4

Recent advances highlighting the role of ferroptosis—induced cell death by iron-dependent lipid peroxidation—suggest that this oxidative microenvironment directly triggers neuroinflammation in depression. Specifically, recent evidence from LPS-elicited neuroinflammation models demonstrates that the downregulation of glutathione peroxidase 4 (GPx4) elevates the susceptibility of microglia to oxidative degradation of lipid and subsequent ferroptosis [49]. Notably, pharmacological or metabolic restoration of GPx4—such as through nicotinamide mononucleotide administration—significantly replenishes GSH pools, suppresses pro-inflammatory cytokine transcription, and ameliorates microglia activation [49]. This intricate coupling between GPx4 depletion and microglial activation sustains a vicious cycle between oxidative stress and neuroinflammation, underscoring the need to expand traditional antioxidant paradigms (e.g., Prdxs and NOS) toward ferroptosis-targeted interventions. Moreover, the modulatory approach of GPx4 should be studied in depression-associated models in the coming years.

### 3.3. Ion Dyshomeostasis

#### 3.3.1. Na^+^, K^+^-ATPase

Na^+^, K^+^-ATPase (NKA), found in all animal cells, is a cellular membrane-localized Na^+^ pump and comprises two distinct subunits: a catalytic α-subunit harboring the ion-transporting domains, and β-subunit, characterized as a monotopic glycoprotein [50,51]. The electrochemical gradient (e.g., Na^+^ and K^+^) is crucial in regulation of ionic homeostasis [52]. In pathological conditions, it has been known that free radicals and NO disrupt NKA activity [53]. In the brain, NKA activity is essential for maintenance of neuronal function, and inhibition of NKA activity by pharmaceutical agents and gene deletion is involved in aging [54], neurological disease including Parkinson’s disease (PD) and Alzheimer’s disease (AD) [55], and mood disorders [56,57]. In rats, stimulation of repeated restraint stress promotes neuroinflammation and oxidative stress by enhancing activator peptide-1, iNOS, and cyclooxygenase (COX)-2 activity, while simultaneously inducing neuronal degeneration and a reduction in α2,3-NKA activity in the frontal cortex, which is an emotional and cognitive region [58]. Additionally, decline in NKA function is further validated in the reserpine-elicited depression rat model, where a substantial decrease in enzyme activity is observed [59]. Briefly, reserpine, known as an antihypertensive agent [60], is used to elicit depression in animal models to assess the pathological manifestations of depressive disorder [61]. In rat models of bipolar disorder, it has been demonstrated that DLBs accompany neuroinflammation following the administration of ouabain, a potent NKA inhibitor [62]. These findings collectively suggest that NKA serves as more than just an ion pump; it acts as a molecular bridge between biochemical stressors and behavioral manifestations. Consequently, the pathoelectrophysiological role of NKA should be highlighted as a fundamental mechanism that links ionic dyshomeostasis and neuroinflammatory cascades in the neuropsychiatric environment. Although direct clinical trials evaluating specific NKA activators in neuropsychiatric disorders are still lacking, growing correlative evidence from brain samples and biomarker studies might support NKA as a viable therapeutic target.

#### 3.3.2. Sarco/Endoplasmic Reticulum Ca^2+^-ATPase 2

The endoplasmic reticulum (ER) is responsible for maintenance of Ca^2+^ homeostasis, modulation of apoptosis, lipid synthesis, and protein folding [63]. When ER Ca^2+^ homeostasis is disrupted, misfolded proteins are accumulated and the unfolded protein response is activated [64]. Persistent ER stress leads to cell death via C/EBP homologous protein (CHOP) and downstream effectors like Casp3 and Casp12 [65,66]. The sarco/endoplasmic reticulum Ca^2+^-ATPase 2 (SERCA2) performs a vital function in ER-Ca^2+^ regulation and pumps cytosolic Ca^2+^ into the ER lumen [67]. SERCA2 dysregulation mediates ER Ca^2+^ depletion, followed by protein misfolding, ER stress, and apoptosis [68]. Diminished ER-released intracellular Ca^2+^ peak is induced through impaired SERCA2-mediated Ca^2+^ reuptake and reduced ER-Ca^2+^ content, subsequently leads to enhanced ER stress in the hippocampal region of STZ-elicited hyperglycemia mouse models and high glucose-treated human neuroblastoma SH-SY5Y cells [69]. Enhanced ER stress by thapsigargin accelerates cell death through enhanced expression of CHOP, cleaved Casp12, and apoptosis markers, such as Bcl2-associated X (Bax) and cleaved Casp3, thereby contributing to the development of DLBs, including anhedonia, despair, and anxiety, in the hippocampal region of STZ-elicited hyperglycemia mouse models and high glucose-treated human neuroblastomas [69]. Thus, as the modulatory strategy on ER stress, SERCA2 activation with tauroursodeoxycholic acid (TUDCA) reduces ER stress and DLBs in STZ-elicited hyperglycemia mouse models [69]. Moreover, TUDCA is currently undergoing clinical evaluation for various neurodegenerative diseases, including amyotrophic lateral sclerosis (ALS), AD, and PD, owing to its neuroprotective effects [70,71].

#### 3.3.3. Synaptojanin-2-Binding Protein

Importantly, the Ca^2+^ dyshomeostasis mediated by SERCA2 dysfunction closely converges with novel structural modulators at the endoplasmic reticulum (ER)–mitochondria interface. Recent studies have identified synaptojanin-2-binding protein (SYNJ2BP) as a crucial regulator of ER–mitochondria tethering and synaptic plasticity in depression models [72]. The downregulation of SYNJ2BP exacerbates Ca^2+^-dependent mitochondrial apoptosis and neuroinflammation via the disruption of the phosphatidylinositol 4,5-bisphosphate (PIP2)/inositol 1,4,5-trisphosphate signaling axis, positioning the SERCA2-SYNJ2BP crosstalk as a potential nexus for stress-induced neurodegeneration [72]. Although clinical translation targeting SYNJ2BP remains unclear, these findings highlight the SERCA2-SYNJ2BP axis as a potential biomarker for stress-induced neurodegenerative disorders.

#### 3.3.4. Endophilin A1

Endophilin A1 (EPA1) is localized in the synaptic terminals involved in synaptic organization such as endocytosis of synaptic vesicle and actin polymerization [73,74]. A recent study showed that the synaptic-associated protein EPA1 expression is enhanced in the hippocampal region of chronic unpredicted mild stress (CUMS)-elicited rodent models of depression [75]. DLBs and neuronal degeneration are reduced by the production of synaptic-associated proteins, comprising mature neuron marker microtubule-associated protein 2, synaptophysin (SYN), brain-derived neurotrophic factor (BDNF), growth-associated protein-43, and postsynaptic density protein 95 (PSD95), which are upregulated following the suppression of EPA1 in CUMS-elicited rodent models of depression [75]. EPA1 expression is associated with intracellular Ca^2+^ contents and ROS production [76]. In the CUMS-elicited rodent model of depression, the levels of Ca^2+^/ROS are attenuated by suppressing EPA1 in the hippocampal region [75]. Moreover, hippocampal inflammation is exacerbated via the upregulation of the EPA1-NLRP1 inflammasome/apoptosis-associated speck-like protein containing a CARD (ASC)/Casp1 signaling axis in a CUMS-induced rodent model of depression [75]. Thus, mitochondrial structure and hippocampal neuronal ultrastructure are damaged through the involvement of EPA1-mediated Ca^2+^-ROS-NLRP1/ASC/Casp1 signaling in CUMS-elicited rodent models of depression [75].

**Table 1:** Effects of signaling proteins in depression and disease-related models presenting depression-like behavioral or neuroinflammatory outcomes.

Signaling Proteins	Category	Variation in Protein Expression or Activity in Depression Models	Effects of Signaling Proteins in Depression Models	Preclinical		Refs.
				In Vitro	In Vivo	
NLRPs	IAoI	↑	DLBs ↑ and neuroinflammation ↑	High glucose-stimulated mouse microglia	STZ-elicited hyperglycemic rodent model	[36]
SARM1	IAoI	↓	Neuroinflammation ↑ and ROS production ↑	SARM1 knockdown mouse neuronal cells	SARM1-deficiency rodent model of depression *	[38]
Prdxs	OICD	↑	Oxidative stress response ↑	LPS-administrated mouse microglia	LPS-elicited rodent model of depression *	[41]
NOS	OICD	↑	Neuroinflammation ↑ and DLBs ↑	-	LPS-elicited rodent model of depression	[48]
GPx4	OICD	↓	Lipid peroxidation ↑ and ferroptosis ↑	-	LPS-elicited neuroinflammation models *	[49]
NKA	IDH	↓	Neuroinflammation ↑	-	Repeated restraint stress-stimulated rat *, reserpine-elicited depression rat model *, ouabain-elicited bipolar disorder rat model *	[58,59,61,62]
SERCA2	IDH	↓	ER-mediated intracellular Ca^2+^ ↓ and ER stress ↑	High glucose-treated human neuroblastoma	STZ-elicited hyperglycemia mouse model *	[69]
SYNJ2BP	IDH	↓	Ca^2+^-dependent mitochondrial apoptosis ↑ and neuroinflammation ↑	-	CUMS-elicited depression models *	[72]
EPA1	IDH	↓	Ca^2+^/ROS levels ↓, inflammation ↑, mitochondrial structure and hippocampal neuronal ultrastructure ↑	-	CUMS-elicited rodent model of depression *	[75]

Abbreviation: The initiators and amplifiers of the inflammation, IAoI; oxidative stress and induction of cell death, OICD; ion dyshomeostasis, IDH; NLRPs, NOD-like receptor pyrin domain-containing proteins; DLBs, depression-like behaviors; STZ, streptozotocin; SARM1, sterile α and TIR motif containing 1; ROS, reactive oxygen species; Prdx, peroxiredoxins; LPS, lipopolysaccharide; NOS, nitric oxide synthase; GPx4, glutathione peroxidase 4; NKA, Na^+^, K^+^-ATPase; SERCA2, sarco/endoplasmic reticulum Ca^2+^-ATPase 2; ER, endoplasmic reticulum; SYNJ2BP, synaptojanin-2-binding protein; CUMS, chronic unpredicted mild stress; EPA1, endophilin A1; ↓: decrease; ↑: increase; *: depression-related disease models without direct evaluation of DLBs.

## 4. Modulation of Neuroinflammation and Therapeutic Approaches

Collectively, the pharmacological agents discussed in this section—ranging from natural-derived phytochemicals to synthetic molecules—converge on a shared therapeutic axis: the attenuation of neuroinflammatory cascades, suppression of cellular stress pathways (e.g., ER stress and ferroptosis), and the restoration of microglial homeostasis. Rather than acting through isolated mechanisms, these diverse compounds functionally counteract neuroinflammation by cooperatively suppressing inflammasome activation, oxidative/nitrosative stress, and BBB dysfunction. The following subsections synthesize how these distinct molecular entities orchestrate multi-target protection in preclinical models of depression and neuroinflammation. Notably, while these compounds broadly target the shared neuroinflammatory pathways described above, most are supported primarily by preclinical evidence; among them, only curcumin and mangiferin have reported clinical outcomes to date. To provide clear translation context, these distinctions in preclinical models and clinical interpretations are categorized and summarized in Table 2.

### 4.1. Doxycycline

The pathophysiology of MDD is mediated by excessive oxidative/nitrosative stress, including overproduced peroxide and NO as well as involvement of the BDNF signaling pathway [20,77,78,79]. Furthermore, downregulated levels of an antioxidant defense, such as GSH content, have been demonstrated in the post-mortem MDD brain [80]. Doxycycline (6-Deoxy-5-hydroxytetracycline) is a tetracycline antibiotic used for treating infections elicited by both Gram-negative and Gram-positive bacteria [81] and possesses various pharmacological functions, such as reduction of oxidative stress [82] and anti-inflammatory properties [83] in the central nervous system (CNS). Neuroinflammation is reduced by treatment with doxycycline in brain area of LPS-elicited rodent models of depression, thereby attenuating DLBs [84]. In addition, treatment with doxycycline induces through enhanced GSH levels an antioxidant effect in the brain area of LPS-elicited rodent models of depression [84].

### 4.2. Curcumin

Curcumin is a bioactive phytochemical derived from *Curcuma longa* and possesses diverse biological fuctions, such as mitigation of inflammation [85], and anti-carcinogenic [86], antioxidant [87], neuroprotective [88], and anti-depressant properties [89]. The neuroinflammation is downregulated through reduced expression of inflammatory cytokines, iNOS, and COX-2 via NF-κB signaling, thereby ameliorating microglial activation and associated behavioral impairments following curcumin treatment in the hippocampal region and prefrontal cortex (PFC) of LPS-elicited rodent models of depression [90,91]. Recent studies have moved beyond the monoamine hypothesis, positioning curcumin as a multi-target agent that simultaneously regulates the hypothalamic–pituitary–adrenal (HPA) axis, neuroinflammation, and oxidative stress pathways to alleviate depressive symptoms [92,93,94]. Additionally, nanoparticle platforms have been applied to enhance its availability. For instance, curcumin-coated iron oxide nanoparticles restore the activity of monoaminergic neurotransmission as well as protect against oxidative stress in reserpine-elicited depression rat models [95]. Consequently, while clinical evidence supports the therapeutic potential of curcumin in reducing depressive symptoms, its utility remains constrained by heterogeneous formulations and limited bioavailability [96,97]. Addressing these challenges through standardized clinical validation and advanced carrier platforms would be crucial to bridge the gap between experimental findings and psychiatric practice.

### 4.3. Mangiferin

Several studies have reported that oxidative/nitrosative stress promotes oxidative degradation of lipid, impaired DNA, decrease in GSH level, and reduced activity of antioxidant enzymes, thereby leading to the pathophysiology of depression and anxiety [17,18,19,20]. Mangiferin, derived from the bark, leaves, and root of *Mangifera indica*, is a major phytochemical that is a natural C-glucosylxanthone [98]. Mangiferin has various biological functions, including reduction of oxidative stress [99], and cardioprotective [100], anti-inflammatory [101], hepatoprotective [102], anti-tumor [103], anti-diabetic [104], and monoamine oxidase inhibition [105] properties. Treatment with mangiferin attenuates neuroinflammation by restoring BDNF expression and enhancing antioxidant activities, including superoxide dismutase (SOD), catalase, and GSH levels in the hippocampal region and PFC, thereby preventing DLBs and anxiety-like behaviors (ALBs) in LPS-elicited rodent models of depression [106]. Additionally, mechanistic investigations have demonstrated that mangiferin suppresses the maturated NLRP3 inflammasome within the hippocampal region, consequently inhibiting the secretion of major pro-inflammatory agents, including IL-1β, which performs a critical role in the pathogenesis of stress-elicited depression [107]. Mangiferin was investigated in postpartum depression, where it was shown to alleviate DLBs by inhibiting MAPK signaling and preventing excessive microglial activation under in vivo and in vitro conditions [108]. In recent double-blind, placebo-controlled trials, extracts of mango leaf (Zynamite^®^ S) acutely improves mood states for example by reducing tension and depression, as well as improves mental clarity in humans [109]. Overall, because human clinical data remain restricted to healthy cohorts receiving crude mango leaf extracts rather than purified mangiferin [109], transitioning from mechanistic preclinical studies to rigorous clinical trials using purified formulations is imperative to establish the therapeutic efficacy and safety of mangiferin in MDD patients.

### 4.4. Etazolate

Several studies reported that etazolate is a selective inhibitor of the PDE4 enzyme and its pharmacological effects include preserving memory loss [110,111,112], and anxiolytic [113] and anti-depressant-like effects [114]. Treatment with etazolate attenuates DLBs, including anhedonia and neuroinflammation, by suppressing the expression of IL-1β and PDE4 isozymes (specifically PDE4A, 4B, and 4D), while simultaneously enhancing cyclic adenosine monophosphate (cAMP)/phospho (p)-cAMP response element-binding protein (CREB)/BDNF signaling in the LPS-elicited rodent model of depression [115]. Furthermore, the application of etazolate to post-traumatic stress disorder models has shown that it effectively prevents memory impairment and ALBs by restoring cAMP-mediated signaling pathways [116]. Additionally, current research trends point toward the ability of etazolate to modulate myelin-related gene expression, offering a strategic opening for treating depression linked to subtle neurodegenerative damage [117]. Taken together, these findings characterize etazolate as a versatile therapeutic candidate capable of modulating the complex interplay between neuroinflammation and structural brain plasticity in the context of MDD.

### 4.5. 1-Methyl-3-(Phenylselanyl)-1H-Indole

Selenium, known as a crucial mineral for human vitality, induces various physiological characteristics, including antioxidant [118], anti-depressant-like [119,120], anti-genotoxic [121], neuroprotective, and anti-apoptotic effects [122]. Neuroinflammation and oxidative stress are decreased through the reduced expression of inflammatory cytokines and iNOS by treatment with 1-methyl-3-(phenylselanyl)-1H-indole (MFSind), an organoselenium compound, in the PFC and hippocampal region of an STZ-elicited diabetic mouse model [31]. In addition, serotonin production is enhanced through the reduced expression of indoleamine-2,3-dioxygenase (IDO) by MFSind treatment in the PFC and the hippocampal region of the STZ-elicited diabetic mouse model [31]. Treatment with MFSind inhibits the increase in blood glucose level through increased insulin receptor substance-1/glucose transporter (GLUT)-4 signaling, thereby suppressing DLBs in the STZ-elicited diabetic mouse model [31].

### 4.6. Fenretinide

Fenretinide (Fen) is a synthesized retinoid 4-hydroxy(phenyl) retinamide and elicits various pharmaceutical effects, such as anti-cancer, anti-inflammatory, and antioxidant effects [123,124,125]. In an LPS-elicited brain injury mouse model, Fen treatment inhibits oxidative stress and neuroinflammation by upregulating the Nrf2 signaling and downregulating the NF-κB, pro-inflammatory agents, and vascular cell adhesion molecule-1 (VCAM-1) [126]. These molecular changes lead to the alleviation of brain impairment and cell death, subsequently attenuating BBB dysfunction through upregulated expression levels of tight junction proteins, comprising occludin and zonula occludens-1, thereby improving brain injury and DLBs in the LPS-elicited brain injury mouse model [126].

### 4.7. Ginsenoside Rb1

Ginsenoside Rb1 (GRb1) is a class of active phytochemical isolated from Ginseng and has been reported to induce various neuropharmaceutical effects, including regulation of monoamine neurotransmitters, maintenance of the HPA axis, and anti-inflammatory, anti-depressant, and neuroprotective activities [127,128,129,130,131]. In chronic mild stress (CMS)-exposed mice and LPS-stimulated primary microglia, treatment with GRb1 stimulates the peroxisome proliferator-activated receptor (PPAR)γ signaling pathway, which inhibits neuroinflammation by stimulating the conversion of microglia from an M1 (pro-inflammatory) to an M2 (an anti-inflammatory)-like phase [132]. Although the dynamic and continuous spectrum of microglial activation driven by local signals extends beyond the classical, rigid “M1/M2” binary framework, this microglial shift also leads to the upregulation of anti-inflammatory agents in the hippocampal region and cortex of CMS-exposed mice and LPS-stimulated primary microglia [132]. Consequently, this modulated microenvironment enhances neurogenesis, thereby alleviating DLBs, including anhedonia, in CMS-exposed rodent models of depression [132].

### 4.8. Sericin

Silk sericin is a natural hydrophilic polymer produced by silkworms [133] and possesses different pharmacological properties including antioxidant [134], anti-tumor [135], anti-inflammatory [136], anti-bacterial [137], and wound healing [138] effects. In the brain, sericin has been reported to be involved in inhibition of neuronal apoptosis in the hippocampal region of rats with type 2 diabetes mellitus [139], reduced oxidative degradation of lipids, and augmentation of enzymatic antioxidant activities in both the brain and peripheral tissues of AD-elicited and oxidative stress-elicited hypercholesterolemic rats [140,141]. Moreover, treatment with sericin inhibits oxidative stress production by enhancing antioxidant activities, including SOD and GPx activity, thereby reducing neuroinflammation through downregulated production of pro-inflammatory agents and NF-κB and increased mitochondrial membrane potential (MMp) level in the PFC and hippocampal region of restraint stress-stimulated depressive (RSSD) rodent models [142]. The restored mitochondrial biology by sericin attenuates apoptosis through enhanced anti-apoptotic signaling, including B-cell lymphoma (Bcl)-2, and suppressed apoptotic signaling, such as Bax, cleaved-Casp9, cleaved-Casp3, and cytosolic cytochrome c in the PFC and hippocampal region of RSSD rodent models [142]. In addition, levels of serum corticosterone (CORT), which contributes to the development and persistence of depressive symptoms [143,144,145], as well as ALBs and DLBs are improved by treatment with sericin in RSSD rodent models [142].

### 4.9. Edaravone

Edaravone (EDA) is used as a scavenger of free radicals and possesses potent pharmacological effects, such as mitigation of inflammation as well as antioxidant and neuroprotective effects [146]. Moreover, several studies have described its therapeutic potential in acute ischemic stroke [147], acute cerebral infarction [148], ALS progression [149], reduction of oxidative stress and mitigation of inflammation effects by the Nrf2/heme oxygenase (HO)-1 signaling pathway in cerebral infarction and bronchial asthma [150], and alleviation of DLBs [151,152]. Treatment with EDA inhibits neuroinflammation by downregulating the TLR4/NF-κB pathway and subsequent inflammatory cytokine expression, thereby attenuating neuronal dysfunction, microglial activation, and astrocyte dysfunction through the augmented expression levels of triggering receptors expressed on myeloid cells 2 (TREM2), connexin (Cx)43, and Cx30 in the hippocampal region and medial PFC (mPFC) of a chronic social defeat stress (CSDS)-stimulated rodent model of depression [153]. TREM2, found in microglia, is a pattern recognition receptor [154,155,156] and has an anti-inflammatory function [157]. In addition, Cx43 and Cx30, mainly found in astrocytes, are astrocytic network proteins that conduct regulation of ion interaction for activation and secretion of signaling modules in astroglial cells [158,159]. In the hippocampal region and mPFC of the CSDS model, oxidative stress-mediated mitochondrial damage is inhibited through upregulated enzymatic antioxidant activities, including SOD and GPx activities, while decreased energy metabolites, such as guanosine monophosphate, guanosine diphosphate, and adenosine monophosphate, are restored by EDA treatment [153]. Consequently, ALBs and DLBs are ameliorated by EDA treatment via the activated Sirtuin1/Nrf2/HO-1/GPx4 pathway in the CSDS-elicited depression model [153].

### 4.10. Fuzi and Ganjiang Extraction

Chinese medicines Fuzi and Ganjiang (FG), isolated from the branch roots of *Aconitum carmichaelii* Debx. and *Zingiber officinale* Rosc. [160], respectively, are used in combination for treating inflammation and heart failure [160,161]. Cancer-related fatigue (CRF) represents a debilitating and unyielding state of physical, psychological, or cognitive exhaustion that directly impairs daily functioning in oncology patients [162]. Various cancer therapies, including radiotherapy, chemotherapy, and hormonal or biological therapies, exacerbate fatigue levels, which could be elevated prior to the onset of cancer therapy [163]. In particular, neuroinflammation-elicited extreme fatigue contributes to exacerbation of depression-like symptoms in patients with CRF [163]. Treatment with FG reduces NO production and prostaglandin (PGE)2 secretion by inhibiting iNOS and COX-2 expression, respectively, thereby attenuating neuroinflammation via downregulated NF-κB signaling and activated Nrf2/HO-1 signaling in LPS-treated mouse microglia [164]. Consistent with in vitro experiments, DLBs are improved through decreased iNOS and COX-2 expression levels by FG treatment in CRF-elicited rodent models of depression [164].

### 4.11. Total Saponins of Panax Ginseng (TSG)

Total saponins of *Panax ginseng* (TSG), as an active phytochemical isolated from Ginseng (*Panax ginseng* C. A. Meyer), induces alleviation of depressive behaviors in patients [165,166,167] and possesses various biological properties, including anti-inflammatory [168] and neuroprotective effects [169]. Treatment with TSG attenuates neuroinflammation through downregulated production of major pro-inflammatory agents, comprising TNF-α, IL-6 and IL-1β, thereby decreasing oxidative stress production through inhibition of the C-X3-C motif chemokine ligand (CX3CL)1/C-X3-C motif chemokine receptor (CX3CR)1/p38/JNK pathway in the hippocampal region of CUMS-elicited depression rat models and LPS-stimulated astrocytes [170]. These molecular alterations induce alleviation of neuronal damage and DLBs by treatment with TSG in the hippocampal region of CUMS-elicited depression rat models [170].

### 4.12. Cardamom Oil

Cardamom (*Elettaria cardamomum* L.), a member of the *Zingiberaceae* family [171], is used to alleviate depression [172,173,174] and anxiety [175], and the active phytochemicals in cardamom oil (CMO) include 1,8 cineole, α-terpinyl acetate, limonene, linalyl acetate, and linalool [176]. Several studies report that CMO possesses antioxidant [177], anti-anxiety [178], anti-bacterial [179], neuroprotective [176], and anti-inflammatory [180] properties. The CMO treatment increases the levels of monoamine neurotransmitters, comprising dopamine (DA), serotonin, and norepinephrine, by inhibiting monoamine oxidase activity, and subsequently attenuates oxidative/nitrosative stress in the hippocampal region and cortex of reserpine-elicited depression rat models [181]. Oxidative stress-reduced activities of NKA and acetylcholinesterase and BDNF levels are restored by treatment with CMO, thereby alleviating DLBs in reserpine-elicited depression rat models [181].

### 4.13. Scutellarin

Scutellarin, found in *Erigeron breviscapus*, is a flavone and major phytochemical and possesses diverse pharmaceutical effects, including anti-fibrosis [182], anti-atherosclerosis [183,184], anti-myocardial ischemia/reperfusion [185], and anti-anxiety [186]. Acute ischemic stroke (AIS) accounts for a substantial proportion of global mortality and long-term functional impairment [187], and the primary effective treatments for AIS patients are thrombolytic therapy and mechanical thrombectomy [188]; however, revascularization leads to critical secondary impairment, known as cerebral ischemia/reperfusion injury (CIRI) [189], which is elicited by the oxygen and glucose deprivation/re-oxygeneration (OGD/R) process applied to neurons [190] and by middle cerebral artery occlusion reperfusion (MCAO/R) applied to in vivo models [191]. Moreover, development of post-stroke depression is associated with CIRI [192]. Treatment with Scutellarin inhibits free radical overload by activating the phosphoinositide 3-kinase (PI3K)/protein kinase B (Akt)/Nrf2/HO-1 axis and SOD activity, and subsequently reduces neuroinflammation through downregulated production of NF-κB and major pro-inflammatory agents, comprising IL-6, TNF-α, and IL-1β in OGD/R-damaged mouse hippocampal neurons and MCAO/R-damaged rat models [193]. Furthermore, neuroprotective effects, including reduction in cerebral infarct volume and BBB permeability, are induced by treatment with Scutellarin, thereby alleviating DLBs in OGD/R-damaged mouse hippocampal neurons and MCAO/R-damaged rat models [193], suggesting that Scutellarin can be considered as a potential anti-depressant drug.

### 4.14. Saikosaponin B2

Saikosaponin B2 (SNB2) is one of the major components found in *Radix Bupleuri* [194]. Numerous studies have reported that SNB2 induces different pharmaceutical effects, including mitigation of inflammation [195], anti-cancer [196], and anti-viral effects [197]. Treatment with SNB2 increases MMp levels and reduces levels of oxidative stress and iron accumulation, thereby alleviating ferroptosis through amplified expression of GPx4, solute carrier family 7 member 11 (SLC7A11), and ferritin heavy chain as well as downregulated expression of acyl-CoA synthetase long chain family member 4 (ACSL4) and transferrin receptor (TFR) in LPS-stimulated primary mouse microglia [198]. The ferroptosis-enhanced ER stress and the Ca^2+^ increase are attenuated through the suppressed expression of ER stress-related factors, comprising X-box binding protein (XBP)1, CHOP, and activating transcription factor (ATF)6, by SNB2 treatment in primary mouse microglia [198]. In addition, neuroinflammation is ameliorated via inhibited TLR4/NF-κB signaling, and subsequently neuronal damage and microglia polarization are reduced by treatment with SNB2 in LPS-stimulated primary mouse microglia and the hippocampal region of CUMS-elicited rodent models of depression [198]. Consequently, DLBs are improved by treatment with SNB2 in CUMS-elicited rodent models of depression [198].

### 4.15. Paeony Glycoside

Total paeony glycoside (TPG), isolated from *Paeonia lactiflora* Pallas, is a bioactive phytochemical and contains paeoniflorin, albiflorin, hydroxyl-paeoniflorin, and benzoylpaeoniflorin [199]. Numerous studies have reported that TPG possesses various pharmacological effects, including mitigation of inflammation [200], liver-protecting [201], and nervous system-protecting properties [202]. Treatment with TPG restores mitochondrial homeostasis in the hippocampal region of a CUMS-elicited rodent model of depression and CORT-stimulated mouse hippocampal neuronal cells by enhancing mitophagy, which is mediated through the upregulation of autophagy-related proteins such as PINK1, Parkin, autophagy-related protein (ATG)5, and the microtubule-associated protein 1A/1B-light chain (LC)3-II/I ratio, alongside autophagosome activation [203]. In addition, recovered mitochondrial biology attenuates maturation of NLRP3 inflammasome by decreasing ROS, and subsequently alleviates pyroptosis through downregulation of pyroptosis-related proteins, such as Casp1, ASC, GSDMD, and IL-1β in the hippocampal region of a CUMS-elicited rodent model of depression and CORT-stimulated mouse hippocampal neuronal cells [203]. Furthermore, altered NLRP3 activation leads to neuroinflammatory inhibition and neuroprotective promotion, thereby alleviating ALBs and DLBs through upregulated neurotransmitter levels and downregulated CORT levels by treatment with TPG in CUMS-elicited rodent models of depression [203].

### 4.16. Auraptene

Auraptene (7-geranyloxycoumarin), as a naturally abundant monoterpene coumarin, is isolated from the Bael fruit (*Aegle marmelos*) and the Seville orange (*Citrus aurantium*), which are members of the *Rutaceae* family [204]. It has been studied that Auraptene possesses diverse pharmacological properties, including anti-cancer [205], anti-depressant [206], and antioxidants effects [207]. Treatment with Auraptene attenuates neuroinflammation by stimulating the conversion of microglia from an M1 (pro-inflammatory) to an M2 (an anti-inflammatory)-like phase, and subsequently leads to downregulation of production of major pro-inflammatory agents, including IL-6, TNF-α, and IL-1β, and upregulation of production of major anti-inflammatory agents, including TGF-β, IL-4, and IL-10 in the hippocampal region of LPS stimulation combined with CUMS-elicited rodent models of depression and LPS-stimulated mouse microglia [208]. In addition, oxidative stress production and phagocytosis are reduced by treatment with Auraptene, thereby improving DLBs through attenuation of serum adrenocorticotropic hormone (ACTH) and CORT levels, which leads to the pathogenesis and maintenance of depressive symptoms [143,144,145] in LPS stimulation combined with CUMS-elicited rodent models of depression [208].

### 4.17. Atraric Acid

Atraric acid is a natural product derived from Linchen which includes various components, such as atranorin, usnic acid, and protolichesterinic acid [209]. Several studies have reported that atraric acid induces various biological effects, including anti-inflammatory [210], antioxidant [211], and anti-cancer properties [212]. The escalating global prevalence of obesity, a chronic metabolic disorder, has positioned it as a major threat to public health. Extended consumption of a high-fat diet (HFD) is consistently identified in the epidemiological literature as a major risk factor for developing obesity, which is associated with various neurological disorders, such as cognitive dysfunction [213]. Diets rich in fatty foods induce the impairment of cognitive functions, including memory, which ultimately heightens the vulnerability of individuals with obesity to neurocognitive deterioration [214]. In addition to the in vivo HFD model, the obesity model is induced by stimulation of oleic acid/palmitic acid (OA/PA) applied in vitro [215]. Treatment with atraric acid enhances autophagy by inhibiting the mechanistic targets of rapamycin (mTOR) activation and NF-κB signaling, thereby scavenging oxidative stress through Nrf2/Kelch-like ECH-associated protein (Keap)1/HO-1 activation and alleviating neuroinflammation in the brain of a HFD mouse model and OA/PA-stimulated mouse hippocampal neuronal cells [216]. In addition, neuroprotective effects are enhanced through the downregulated expression of S100 Ca^2+^-binding protein B (S100B) and neuron-specific enolase, which are brain injury biomarkers, and cognitive dysfunction, as well as ALBs and DLBs are subsequently alleviated by treatment with atraric acid in HFD mouse models [216].

### 4.18. Chlorogenic Acid

Chlorogenic acid (CGA), a polyphenolic phytochemical, is derived from coffee, fruits, and vegetables [217]. Several studies have reported that CGA possesses functions including reduction of oxidative stress [218], mitigation of inflammation [219], and neuroprotective properties [220]. Treatment with CGA reduces the CORT–glucocorticoid receptor (GR) interaction, thereby scavenging oxidative stress production in the hippocampal region of a chronic stress (CS)-elicited rodent model of depression and dexamethasone (DXM)-stimulated rat microglia [221]. In addition, NLRP3 inflammasome and microglia activation are inhibited, which subsequently suppresses neuroinflammation through PI3K/Akt/Nrf2 activation by treatment with CGA in the hippocampal region of CS-elicited depression rat models and DXM-stimulated rat microglia [221]. Consequently, neuroprotective effects are enhanced and DLBs are improved by treatment with CGA in CS-elicited depression rat models [221].

**Table 2:** Mechanisms of compounds in depression and disease-related models presenting depression-like behavioral or neuroinflammatory outcomes.

Compounds	Related Molecular Mechanism	Biological Effects in Depression Models	Preclinical		Clinical Interpretation	Refs.
			In Vitro	In Vivo		
Doxycycline	GSH level ↑	Neuroinflammation ↓, antioxidants ↑, and DLBs ↓	-	LPS-elicited rodent model of depression	-	[84]
Curcumin	Expression of inflammatory cytokine, iNOS, and COX-2 ↓	Neuroinflammation ↓, DLBs ↓, and antioxidants ↑	-	LPS-elicited rodent model of depression and reserpine-elicited depression rat model	Alleviation of depressive symptoms in MDD patients	[90,91,92,93,94,95,96,97,222,223]
Mangiferin	BDNF expression ↑, SOD ↑, catalase ↑, and GSH levels ↑, NLRP3 inflammasome activation ↓, IL-1β and MAPK ↓	Neuroinflammation ↓, antioxidants ↑, and DLBs and ALBs ↓	-	LPS-elicited rodent model of depression	Improvement of mood states in healthy individuals	[106,107,108,109]
Etazolate	IL-1β and PDE4 isozymes ↓, cAMP/p-CREB/BDNF signaling ↑, cAMP-mediated signaling pathways ↑	Neuroinflammation and DLBs ↓, memory impairment and ALBs ↓	-	LPS-elicited rodent model of depression and post-traumatic stress disorder models	-	[115,116]
MFSind	Expression of inflammatory cytokines and iNOS ↓, expression of IDO ↓, insulin receptor substance-1/GLUT-4 signaling ↑	Neuroinflammation ↓, oxidative stress ↓, and DLBs ↓	-	STZ-elicited diabetic mouse model	-	[31]
Fen	Nrf2 signaling ↑, NF-κB, VCAM-1, and pro-inflammatory agents ↓, and expression of ZO-1 and occludin ↑	Oxidative stress ↓, neuroinflammation ↓, brain impairment ↓, BBB dysfunction ↓, and DLBs ↓	-	LPS-elicited brain injury mouse model	-	[126]
GRb1	PPARγ pathway ↑ and anti-inflammatory cytokines ↑	Neuroinflammation ↓, neurogenesis ↑, and DLBs ↓	LPS-stimulated primary microglia	CMS-exposed mice	-	[132]
Sericin	Activity of SOD and GPx ↑, expressions of NF-κB and inflammatory cytokine ↓, MMp level ↑, Bcl-2 ↑, Bax ↓, cleaved-Casp9 ↓, cleaved-Casp3 ↓, and cytosolic cytochrome c ↓	Oxidative stress ↓, neuroinflammation ↓, apoptosis ↓, and ALBs and DLBs ↓	-	Rats with type 2 diabetes mellitus, AD-elicited and oxidative stress-elicited hypercholesterolemic rats, and RSSD rodent model	-	[139,140,141,142]
EDA	TLR4/NF-κB pathway ↓, inflammatory cytokine expression ↓, expression of TREM2, Cx43, and Cx30 ↑, activity of SOD and GPx ↑, and Sirtuin1/Nrf2/HO-1/GPx4 pathway ↑	Neuroinflammation ↓, neuronal dysfunction ↓, microglial activation ↓, astrocyte dysfunction ↓, oxidative stress-mediated mitochondrial damage ↓, and ALBs and DLBs ↓	-	CSDS-stimulated rodent model of depression	-	[153]
FG	Expression of iNOS and COX-2 ↓, NF-κB signaling ↓, and Nrf2/HO-1 signaling ↑	Neuroinflammation ↓ and DLBs ↓	LPS-treated mouse microglia	CRF-elicited rodent model of depression	-	[164]
TSG	Expression of IL-1β, IL-6, and TNF-α ↓ and CX3CL1/CX3CR1/p38/JNK pathway ↓	Neuroinflammation ↓, oxidative stress production ↓, neuronal damages ↓, and DLBs ↓	LPS-stimulated astrocytes	CUMS-elicited depression rat model	-	[170]
CMO	The levels of serotonin, norepinephrine, and DA ↑ and BDNF levels ↑	Oxidative/nitrosative stress ↓ and DLBs ↓	-	Reserpine-elicited depression rat model	-	[181]
Scutellarin	PI3K/Akt/Nrf2/HO-1 signaling axis ↑, SOD activity ↑, and expression of NF-κB, TNF-α, IL-1β, and IL-6 ↓	Oxidative stress ↓, neuroinflammation ↓, neuroprotective effects ↑, and DLBs ↓	OGD/R-damaged mouse hippocampal neuronal cells	MCAO/R-damaged rat model	-	[193]
SNB2	Expression of SLC7A11, ferritin heavy chain, and GPx4 ↑, expression of ACSL4 and TFR ↓, expression of CHOP, ATF6, and XBP1 ↓, and TLR4/NF-κB signaling ↓	MMp levels ↑, oxidative stress ↓, iron accumulation ↓, ferroptosis ↓, ER stress ↓, Ca^2+^ increase ↓, neuroinflammation ↓, DLBs ↓	LPS-stimulated primary mouse microglia	CUMS-elicited rodent model of depression	-	[198]
TPG	PINK1 ↑, Parkin ↑, ATG5 ↑, LC3-II/I ratio ↑, autophagosome ↑, NLRP3 inflammasome activation ↓, ASC ↓, Casp1 ↓, GSDM ↓, IL-1β ↓, neurotransmitter levels ↑, and CORT levels ↓	Mitochondrial homeostasis ↑, pyroptosis ↓, neuroinflammatory ↓, and neuroprotective ↑	CORT-stimulated mouse hippocampal neuronal cells	CUMS-elicited rodent model of depression *	-	[203]
Auraptene	TNF-α ↓, IL-1β ↓, IL-6 ↓, IL-4 ↑, IL-10 ↑, TGF-β ↑, and serum levels of ACTH and CORT ↓	Neuroinflammation ↓, oxidative stress ↓, phagocytosis ↓, and DLBs ↓	LPS-stimulated mouse microglia	LPS stimulation combined with CUMS-elicited rodent model of depression	-	[208]
Atraric acid	mTOR activation ↓, NF-κB signaling ↓, Nrf2/Keap1/HO-1 activation ↑, and expression of S100B and neuron-specific enolase ↓	Autophagy ↑, oxidative stress ↓, neuroinflammation ↓, and neuroprotective effects ↑	OA/PA-stimulated mouse hippocampal neuronal cells	HFD mouse model *	-	[216]
CGA	GR interaction ↓, NLRP3 inflammasome ↓, PI3K/Akt/Nrf2 activation ↑	Oxidative stress production ↓, neuroinflammation ↓, neuroprotective effects ↑, and DLBs ↓	DXM-stimulated rat microglia	CS-elicited depression rat model	-	[221]

Abbreviation: GSH, glutathione; DLBs, depression-like behaviors; LPS, lipopolysaccharide; iNOS, inducible nitric oxide synthase; COX, cyclooxygenase; BDNF, brain-derived neurotrophic factor; SOD, superoxide dismutase; NLRP, NOD-like receptor pyrin domain-containing protein; IL, interleukin; MAPK, mitogen-activated protein kinase; ALBs, anxiety-like behaviors; PDE, phosphodiesterase; cAMP, cyclic adenosine monophosphate; p-CREB, phospho-cAMP response element-binding protein; MFSind, 1-methyl-3-(phenylselanyl)-1H-indole; IDO, indoleamine-2,3-dioxygenase; GLUT, glucose transporter; STZ, streptozotocin; Fen, fenretinide; Nrf2, nuclear factor erythroid 2-related factor; NF-κB, nuclear factor kappa-light-chain-enhancer of activated B cells; VCAM, vascular cell adhesion molecule; ZO-1, zonula occludens-1; BBB, blood–brain barrier; GRb1, ginsenoside Rb1; PPAR, peroxisome proliferator-activated receptor; CMS, chronic mild stress; GPx, glutathione peroxidase; MMp, mitochondrial membrane potential; Bcl, B-cell lymphoma; Bax, Bcl2-associated X; Casp, caspase; AD, Alzheimer’s disease; RSSD, restraint stress-stimulated depressive; EDA, edaravone; TLR, toll-like receptor; TREM, triggering receptor expressed on myeloid cells; Cx, connexin; HO, heme oxygenase; CSDS, chronic social defeat stress; FG, Fuzi and Ganjiang; CRF, cancer-related fatigue; TSG, total saponins of *Panax ginseng*; TNF, tumor necrosis factor; CX3CL, C-X3-C motif chemokine ligand; CX3CR, C-X3-C motif chemokine receptor; JNK, c-Jun N-terminal kinase; CUMS, chronic unpredicted mild stress; CMO, cardamom oil; DA, dopamine; PI3K, phosphoinositide 3-kinase; Akt, protein kinase B; OGD/R, oxygen and glucose deprivation/re-oxygeneration; MCAO/R, middle cerebral artery occlusion/reperfusion; SNB2, saikosaponins B2; SLC7A11, solute carrier family 7 member 11; ACSL, acyl-CoA synthetase long chain family member; TFR, transferrin receptor; CHOP, c/ebp homologous protein; ATF, activating transcription factor; XBP, x-box binding protein; ER, endoplasmic reticulum; TPG, total paeony glycoside; PINK, phosphatase and tensin homolog-induced kinase; ATG, autophagy-related protein; LC, microtubule-associated protein 1A/1B-light chain; ASC, apoptosis-associated speck-like protein containing a CARD; GSDM, gasdermin; ACTH, adrenocorticotropic hormone; CORT, corticosterone; mTOR, mechanistic target of rapamycin; Keap, kelch-like ech-associated protein 1; S100B, S100 Ca^2+^-binding protein B; HFD, high-fat diet; OA/PA, oleic acid/palmitic acid; GR, glucocorticoid receptor; CS, chronic stress; DXM, dexamethasone; ↓: decrease; ↑: increase; *: depression-related disease models without direct evaluation of DLBs or ALBs.

## 5. Clinical Perspectives and Future Directions

While the preclinical evidence reviewed herein underscores the therapeutic potential of various synthetic and natural-derived agents in mitigating neuroinflammation-driven depressive behaviors, several critical gaps should be addressed to translate these findings from bench to bedside. Although these pharmacological agents have demonstrated therapeutic efficacy in attenuating neuroinflammatory and molecular pathology as well as behavioral deficits in various mouse models of depression and disease-related models, clinical translation remains a paramount challenge. Specifically, there is an urgent need to identify and isolate highly specific, periphery-accessible biomarkers from patient blood samples (such as circulating cytokines, oxidative stress markers, or exosomal signaling proteins) that directly reflect the central neuroinflammatory status.

The translational challenge is demonstrated by the difficulties encountered in human clinical trials over the past decade. Numerous studies evaluating repurposed anti-inflammatory agents, such as COX-2 inhibitors (e.g., celecoxib), antibiotics (e.g., minocycline), and TNF-α antagonists (e.g., infliximab), as adjunctive therapies for MDD have yielded rather heterogeneous and inconsistent results [224,225,226,227]. A major caveat regarding this heterogeneity involves methodological limitations, where depressed individuals were recruited based on conventional, symptom-based clinical phenotypes, rather than being adequately stratified based on verified neuroinflammatory markers.

The barrier to biotypic stratification stems from the intrinsic limitations of standard peripheral immune measures. Conventional systemic inflammatory markers often lack CNS specificity, suggesting that peripheral immune profiles may not accurately reflect the localized, low-grade intracellular neuroinflammatory cascades occurring within the CNS glial networks [228,229]. This compartmentalized neuroinflammation underscores the necessity of identifying more proximal, CNS-specific biomarkers, such as neural- or glial-derived extracellular vesicles, to accurately assess and quantify the central neuroinflammatory status and improve the immunophenotypic stratification of patients [230].

To bridge the preclinical-to-clinical translational gap, advanced molecular imaging techniques, particularly positron emission tomography (PET) imaging targeting neuroinflammation, represent a vital modality for in vivo validation in the human brain [231,232]. Meta-analysis of the PET literature shows distinct neuroinflammatory changes in MDD patients, with significant increases in specific cortical and limbic regions, such as the anterior cingulate cortex, PFC, temporal cortex, insula, and hippocampal region [228,233]. While 18 kDa translocator protein (TSPO) has thus far been the primary target for neuroinflammation PET imaging, current in vivo TSPO imaging lacks cellular and functional specificity due to the ubiquitous, multicellular expression of TSPO across microglia, astrocytes, and endothelial cells [234]. Furthermore, conventional TSPO ligands fail to distinguish between pro- and anti-inflammatory phenotypes, precluding the in vivo tracking of their dynamic functional shifts [235]. To overcome these limitations, future efforts should focus on developing novel radiotracers targeting molecular markers of specific cellular and functional states, thereby enabling the quantification of cellular neuroinflammatory dynamics. Such advancements will ultimately facilitate the precise evaluation of targeted therapeutics.

**Figure 2:**
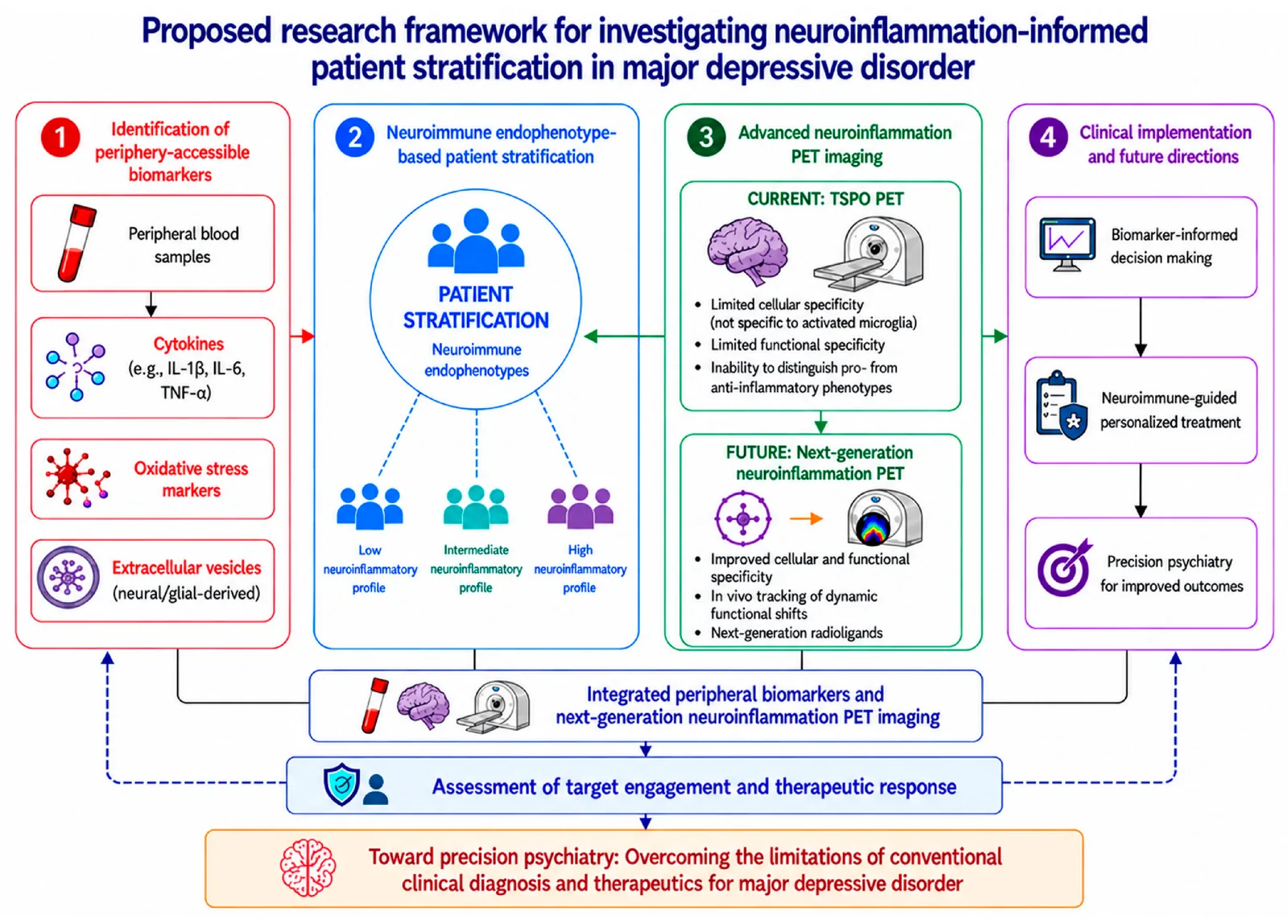
Proposed research framework for investigating neuroinflammation-informed patient stratification in major depressive disorder. This schematic presents a conceptual framework for future research on neuroinflammation-related heterogeneity in MDD. The proposed framework illustrates the integration of periphery-accessible biomarkers, including circulating cytokines, oxidative stress markers, and neural- or glial-derived extracellular vesicles, with clinical information and neuroinflammation PET imaging. Such multimodal integration may facilitate investigations of biologically informed MDD subgroups, target engagement, and therapeutic response. However, peripheral inflammatory markers have limited specificity for central nervous system inflammation, and currently available TSPO PET imaging lacks sufficient cellular and functional specificity. Therefore, this framework should not be interpreted as an established clinical strategy for patient stratification or individualized treatment selection. Prospective and longitudinal studies are required to establish its reproducibility, predictive validity, and clinical utility. Abbreviations: MDD, major depressive disorder; PET, positron emission tomography; IL, interleukin; TNF-α, tumor necrosis factor-α; TSPO, translocator protein.

Given the current limitations of both peripheral inflammatory markers and TSPO PET imaging, the integration of these measures should be regarded as a conceptual research framework rather than an established clinical strategy for patient stratification. Research frameworks such as the Research Domain Criteria have encouraged the investigation of biologically informed dimensions [236], while the emerging field of immunoneuropsychiatry has highlighted the potential relevance of immune-related heterogeneity across neuropsychiatric disorders [237]. Moreover, a recent meta-analysis suggests that the therapeutic efficacy of anti-inflammatory interventions in depressed individuals is contingent on baseline inflammation status [238]. Nevertheless, these findings do not establish that currently available inflammatory biomarkers can reliably identify treatment-responsive subgroups or guide treatment selection for individual patients with MDD. Prospective and longitudinal studies in well-characterized clinical cohorts are therefore required to determine the reproducibility, predictive validity, added clinical value, and treatment-related utility of the proposed approach. If validated, the integration of peripheral biomarkers, clinical characteristics, and next-generation neuroinflammation imaging may eventually support the identification of biologically informed MDD subgroups. Ultimately, shifting the paradigm toward immunophenotypically defined patient stratification may offer a promising avenue to overcome the limitations of conventional therapeutics for MDD and address unmet clinical needs. This proposed framework is illustrated in Figure 2.

## Data Availability

No new data were created or analyzed in this study. Data sharing is not applicable to this article.
